# Chlorhexidine-coated surgical gloves influence the bacterial flora of hands over a period of 3 hours

**DOI:** 10.1186/s13756-018-0395-0

**Published:** 2018-09-06

**Authors:** Miranda Suchomel, Markus Brillmann, Ojan Assadian, Karen J. Ousey, Elisabeth Presterl

**Affiliations:** 1 Institute for Hygiene and Applied Immunology, Medical University of Vienna, Kinderspitalgasse 15, 1090 Vienna, Austria; 2 Department for Hospital Epidemiology and Infection Control, Medical University of Vienna, Vienna, Austria; 3 Institute for Skin Integrity and Infection Prevention, University of Huddersfield, Huddersfield, UK

**Keywords:** Surgical glove, Perforation, Bacterial migration, Antimicrobial efficacy, Surgical site infection, Bacterial skin flora, Antimicrobial glove, Chlorhexidine, Antiseptic

## Abstract

**Background:**

The risk of SSI increases in the presence of foreign materials and may be caused by organisms with low pathogenicity, such as skin flora derived from hands of surgical team members in the event of a glove breach. Previously, we were able to demonstrate that a novel antimicrobial surgical glove coated chlorhexidine-digluconate as the active ingredient on its inner surface was able to suppress surgeons’ hand flora during operative procedures by a magnitude of 1.7 log_10_ cfu/mL. Because of the clinical design of that study, we were not able to measure the full magnitude of the possible antibacterial suppression effect of antimicrobial gloves over a full 3 h period.

**Methods:**

The experimental procedure followed the method for assessment of the 3-h effects of a surgical hand rub’s efficacy to reduce the release of hand flora as described in the European Norm EN 12791. Healthy volunteers tested either an antimicrobial surgical glove or non-antimicrobial surgical latex gloves in a standardized laboratory-based experiment over a wear time of 3 h.

**Results:**

Wearing antimicrobial surgical glove after a surgical hand rub with 60% (*v*/v) n-propanol resulted in the highest 3-h reduction factor of 2.67 log_10_. Non-antimicrobial surgical gloves demonstrated significantly lower (*p* ≤ 0.01) 3-h reduction factors at 1.96 log_10_ and 1.68 log_10_, respectively. Antibacterial surgical gloves are able to maintain a sustainable bacterial reduction on finger tips in a magnitude of almost 3 log_10_ (log_10_ 2.67 cfu) over 3 h wear time.

**Conclusion:**

It was demonstrated that wear of an antibacterial surgical glove coated with chlorhexidine-digluconate is able to suppress resident hand flora significantly over a period of 3-h.

## Background

Surgical site infections (SSIs) constitute a large proportion of all Healthcare Associated Infections (HAI). Overall, at least one of every 20 patients undergoing open surgery will develop an SSI [1, 2]. SSIs are associated with one third of post-operative related deaths [3], but more frequently they may cause cosmetically unacceptable scars, pain, prolonged duration of hospitalization, and emotional stress to patients, relatives, and care givers [4, 5].

SSI rates are influenced by multiple clinical risk factors. However, to cause any SSI, microorganisms will need to contaminate the sterile surgical site. Bacteria involved in SSIs include patients’ own endogenous flora, and those that may be introduced from the environment including the microbial flora of the operating surgical team members [6, 7]. The risk of SSI increases in the presence of foreign materials and may even be caused by organisms with low pathogenicity, such as skin flora derived from hands of surgical team members in the event of a glove breach [8].

Breach of glove integrity may cause bacterial migration from the surgeon’s hand to the surgical site [9]. Therefore, various tactics have been developed to reduce the risk of surgical site contamination with bacteria originating from the surgical team’s hands. The most important measure is preoperative surgical hand antisepsis using an antimicrobial soap (surgical scrub) or an alcohol-based hand rub (surgical rub), which is regarded as standard practice to decrease the microbial bio-burden on surgeons’ hands [10]. However, preoperative surgical hand antisepsis can reduce, but not eradicate the resident flora on the surgeon’s hands [11, 12], and re-grown skin flora therefore still may enter the surgical site in the event of a glove breach [13].

In a previous randomized clinical trial [14] we were able to demonstrate that after vascular surgical procedures involving carotid endarterectomy, peripheral bypass surgery, or revascularization of the common femoral and profunda femoris arteries the frequency of glove perforation was 14% at the end of the interventions. Furthermore, we could demonstrate that the mean number of bacterial colony forming units (cfu) retrieved from the inner layer of intact surgical gloves was 299 cfu/mL after a mean operating time of 112 min. Finally, we could show that a novel antimicrobial surgical glove coated with a complex formulation of 14 ingredients, including chlorhexidine-digluconate (CHG) as the active ingredient on its inner surface was able to suppress surgeons’ hand flora during operative procedures by a magnitude of 1.7 log_10_ cfu/mL.

However, because of the design of that study, we were not able to measure the full magnitude of the possible antibacterial suppression effect of antimicrobial gloves over a full 3 h period. Therefore, the aim of this laboratory-based standardized experimental study following the European Norm (EN) 12,791 [15], the in vivo laboratory assay for testing the bactericidal efficacy of pre-surgical hand preparations, was to close this gap.

## Methods

The experimental procedure followed the method for assessment of the 3-h effect of a surgical hand rub’s efficacy to reduce the release of hand flora as described in the European Norm EN 12791 [15]. The study was conducted at the Institute for Hygiene and Applied Immunology, Medical University, Vienna, Austria. The laboratory was accredited according to EN ISO/IEC 17025:2005 [16] and recognized by the national accreditation body “Akkreditierung Austria”. All areas of testing were approved and reported to the Federal Ministry of Science, Research and Economy, Austria. Approval for this laboratory based experimental work was obtained together with a previously published randomized controlled trial (RCT; ISRCTN 71391952) from the ethics committee of the municipality of Vienna (EK 11–201-1111), and written informed consent was obtained from all participating volunteers.

Twenty-one healthy volunteers tested either an antimicrobial surgical glove (Glove A; Gammex PF with AMT; Ansell Ltd., Richmond, Australia) made of latex or one of the following non-antimicrobial surgical latex glove types: Sempermed Supreme (Glove B; Semperit, Ternitz, Austria) or Gammex PF (Glove C; Gammex PF; Ansell Ltd., Richmond, Australia) randomly allocated to their dominant and non-dominant hand.

The proof of a non-existent antimicrobial property of the uncoated control gloves B and C was carried out in accordance with Annex B of the European Norm EN 12791 [15].

A Latin-square design was used with 3 test groups (glove A and B; glove B and C; glove C and A), each of 7 randomly allotted participants. In each test run all 3 test groups were tested concurrently. At the end of the whole test series each volunteer had used each glove combination (A/B; B/C; C/A) once. Each test run was performed strictly on a Monday in order to allow re-growth of the normal skin flora before the next test run. Hence, after 3 weeks, a total of 42 results were available for each type of glove.

Before each test, every participant washed hands in a standardized manner with non-medicated soap (APOCA; Vienna, Austria) for 1 min as described in EN 12791 [15]. Hands were dried with clean hand towels. Thereafter, fingertips were rubbed and kneaded for 1 min at the base of a petri dish (∅ 9 cm) containing 10 mL tryptic soy broth (TSB; Caso broth®, Merck) for measurement of bacterial pre-values. Subsequently, hand antisepsis was performed using minimum 3 mL reference alcohol 60% (*v*/v) n-propanol (pro analysi, Merck) for 3 min [15]. The bactericidal efficacy of this reference alcohol recommended by the European Norm EN 12791 was demonstrated before [17]. After the alcohol had evaporated, the volunteer donned two different sterile surgical gloves on both hands (A and B, B and C or C and A). Instead of the conditions of the EN 12791 which requires comparing the reduction factor of a test hand rub against a reference product immediately and after 3 h in pre-trained volunteers, the viable log_10_ cfu/mL means of the post-values obtained from the participants’ finger tips [15] of the three groups were compared against each other only after 3 h. During the 3-h phase the participants followed the standard procedure according to the used EN 12791, which states that they shall use their gloved hands as usually simulating a surgery. In case of glove perforation the participant has to be excluded.

After 3 h gloves were donned by a second person without contamination and finger tips of both hands were massaged in petri dishes - one for each hand - filled with 10 mL of a validated neutralizer (90 g/L polysorbate 80, 9 g/L lecithin, and 3 g/L histidine and TSB) active against chlorhexidine [15], and gently massaged for 1 min; quantitative surface cultures were prepared on Tryptone soya agar plates (TSA plates; Caso agar®, Merck) using a sterile pipette tip and a sterile spreaders from all sampling solutions and their decimal dilutions. The agar plates were incubated for up to 48 h at 36 °C ± 1 °C. After incubation, the colony forming unit (cfu) per mL was counted and recorded for each dilution step. The number of cfu per mL sampling fluid was calculated by multiplying the plate count by the dilution factor. In addition to recording cfu/mL counts, viable counts were transformed to decimal logarithms, where appropriate. For computational reasons, values of “0” (log_10_ 0 = −∞) was set at “1” (log_10_ 1 = 0).

### Statistical analysis

Logarithmic reduction factors (log_10_ RFs) were calculated as the intra-individual difference of log_10_ pre-treatment values minus log_10_ post-treatment values after 3 h for each glove type separately. Log_10_ RFs were expressed as means ± standard deviation (±SD), with 95% confidence intervals (CIs) and range. Mean log_10_ RFs were tested for statistical significant difference between the tested groups (A/B; B/C; C/A) by using a paired two-tailed T-test. Negative values were corrected to positive values, if applicable. All tests for significance were run as two-sided tests with alpha was set at the 5% level.

## Results

No significant differences were found between the means of the pre-treatment bacterial counts in any of the experimental test runs (data not shown). The means ranged between 4.39 and 4.55 log_10_ and therefore fulfilled the EN 12791 which requires pre-treatment values higher than 3.5 log_10_. After surgical hand antisepsis using 60% *v*/v n-propanol and a 3 h wear time of glove A (antimicrobial surgical glove) the log_10_ reduction was 2.67, and in the standard surgical gloves log_10_ 1.96 (glove B) and log_10_ 1.68 (glove C), respectively. Overall, after 3 h of wear, the antimicrobial surgical glove (glove A) demonstrated a higher log_10_ reduction, while the non-antimicrobial surgical gloves (glove B, glove C) showed a lower log_10_-reduction (Table 1).

**Table 1:** Mean log_10_ reduction factors after 3 h wear time of three different surgical gloves

Group	N	Mean log_10_ RF	± SD	95%-Confidence Interval (CI)		Min.	Max.
				Lower	Upper		
A	42	2.67	1.24	2.28	3.06	−1.19	5.37
B	42	1.96	1.31	1.55	2.37	−0.14	5.06
C	42	1.68	1.09	1.33	2.02	−0.23	3.85
Total	126	2.10	1.28	1.88	2.33	−1.19	5.37

*RF* reduction factor, *SD* Standard Deviation, *N* sample size, Group A: antimicrobial surgical glove; Group B and group C: non-antimicrobial standard surgical gloves

The difference in the mean log_10_-reduction factors between antimicrobial surgical gloves and non-antibacterial gloves B or C was statistically significant (log_10_ reduction factor 0.71 and 0.99, respectively; *p* = 0.001 and *p* < 0.001, respectively). There was no statistical significant difference in the log_10_ reduction factors between the two non-antimicrobial surgical gloves after a 3 h wear time (log_10_ reduction factor 0.28; *p* = 0.056, Table 2).

**Table 2:** Pair-wise ANOVA comparing differences in log_10_ RFs between glove types

	Paired differences				T	df	Significance
	Mean log_10_ RF	± SD	95%-Confidence Intervals of differences				
			Lower	Upper			
A – B	0.71	1.38	0.28	1.14	3.33	41	0.001
B – C	0.28	1.14	−0.07	0.64	1.62	41	0.056
C – A	0.99	1.20	0.62	1.37	5.37	41	0.000

*df* Degree of freedom, *T* Effect size for statistical test, *SD* Standard Deviation

These results demonstrate that antibacterial surgical gloves were able to maintain a sustainable bacterial reduction on hands in a magnitude of almost 3 log_10_ (log_10_ 2.67 cfu) over 3 h wear time.

## Discussion

SSI rates are influenced by multiple clinical variables. Nonetheless, bacteria may origin from a patient endogenously or may enter the sterile surgical site exogenously from the environment including the microbial flora of the surgical team, particularly in case of glove breach [8, 9, 14, 18]. The risk of glove defects is related to the type of surgery performed, ranging from 7% in urological surgery and 65% in cardio-thoracic surgery [19–23].

Previously, we were able to demonstrate that even after surgical hand antisepsis surgeons may harbour (again) between log_10_ 2.51 cfu to log_10_ 2.72 cfu of bacteria on their fingertips after 3 h wear time of non-antibacterial surgical gloves [14]. The aim of this study was to investigate a possibly present or absent suppressing effect of an antibacterial surgical glove in comparison to non-antibacterial surgical gloves on the skin flora after surgical hand treatment with the reference alcohol 60% (*v*/v) n-propanol of the European Norm EN 12791 [15], the European in vivo laboratory assay for testing bactericidal efficacy of surgical hand treatments, under standardized and reproducible laboratory conditions. Thus, in the present study, we were further able to demonstrate that wear of an antibacterial surgical glove coated with chlorhexidine-digluconate is able to suppress resident hand flora significantly over a period of 3 h and to maintain a sustainable bacterial reduction on hands in a magnitude of almost 3 log_10_ (log_10_ reduction factor 2.67 cfu).

Our study has a number of limitations. First, we were not able to state in exact numbers what happened immediately after surgical hand antisepsis and donning gloves. Second, we were also not able to state how many minutes of wearing an antimicrobial glove would have been needed to observe the first significant difference in cfu counts as compared to a non-antimicrobial glove. These questions can only be answered by conducting a bacterial elimination kinetic study with different measure points as compared to the strict time points required by the European reference method EN 12791. Although our study design would have been able to serve as basis for such an investigation on bacterial kinetics under antibacterial and non-antimicrobial gloves, it would require different sampling time points.

Interestingly, also in the standard glove groups we observed low bacterial counts on fingertips. In theory, the number of cfu on the hand donned with a non-antimicrobial glove should increase over time, while the number of cfu on antibacterially donned hands should remain low or increase only in minute counts. Therefore, the longer an antibacterial glove is worn, the larger the difference in cfu should be. However, by ascertaining that all tested groups were measured at the identical time, a possible influence based on such mechanisms may be ruled out.

Finally, when a new technology, drug, method or other procedure is introduced, it will be expected that a benefit is demonstrated with its use. Clearly, in case of antimicrobial devices, the primary intention to use this is prevention or treatment of infection. Therefore, it is logical that demonstration of prevention or treatment success is scientifically produced. However, concurrently with increasing awareness for infection control and implementation of bundle measures to decrease the burden of infection, demonstration of the clinical efficacy of antimicrobial devices is becoming also increasingly difficult because of the decreasing number of infection in individual surgical procedures. The required size of such randomized clinical trials automatically prohibits and attempt for such studies. If the efficacy of antimicrobial devices needs to be demonstrated clinically, one option would be to conduct such studies during episodes of highly increased incidences of SSI, such as during outbreak situations. Aside of the fact that outbreaks are rarely predictable and timely planning is impossible, the result of a randomized controlled trial performed in such a situation would not allow drawing conclusions for a device’s efficacy in a normal patient population, and any effects would be subject to justified critique. Therefore, the only other two alternatives seem to be the establishment of huge international registries with accepted definitions for SSI, or well-designed experimental clinical or in-vivo studies to evaluate and compare these concepts, preferably under the same test conditions and test methodology.

## Conclusion

In conclusion, it was demonstrated that wear of an antibacterial surgical glove coated with chlorhexidine-digluconate is able to suppress resident hand flora significantly over a period of 3-h.

## References

[1] Smyth ET, McIlvenny G, Enstone JE, Emmerson AM, Humphreys H, Fitzpatrick F (2008). Four country healthcare associated infection prevalence survey 2006: overview of the results. J Hosp Infect.

[2] Prospero E, Cavicchi A, Bacelli S, Barbadoro P, Tantucci L, D’Errico MM (2006). Surveillance for surgical site infection after hospital discharge: a surgical procedure-specific perspective. Infect Control Hosp Epidemiol.

[3] Astagneau P, Rioux C, Golliot F, Brückner G, INCISO Network Study Group (2001). Morbidity and mortality associated with surgical site infections: results from the 1997–1999 INCISO surveillance. J Hosp Infect..

[4] Bayat A, McGrouther DA, Ferguson MW (2003). Skin scarring. Br Med J.

[5] McGarry SA, Engemann JJ, Schmader K (2004). Surgical-site infection due to staphylococcus aureus among elderly patients: mortality, duration of hospitalization and cost. Infect Control Hosp Epidemiol.

[6] Allegranzi B, Bischoff P, de Jonge S, Kubilay NZ, Zayed B, Gomes SM (2016). New WHO recommendations on preoperative measures for surgical site infection prevention: an evidence-based global perspective. Lancet Infect Dis.

[7] Berríos-Torres SI, Umscheid CA, Bratzler DW, Leas B, Stone EC, Kelz RR (2017). Centers for Disease Control and Prevention guideline for the prevention of surgical site infection, 2017. JAMA Surg.

[8] Misteli H, Weber WP, Reck S, Rosenthal R, Zwahlen M, Fueglistaler P (2009). Surgical glove perforation and the risk of surgical site infection. Arch Surg.

[9] Hübner NO, Goerdt AM, Stanislawski N, Assadian O, Heidecke CD, Kramer A, Partecke LI (2010). Bacterial migration through punctured surgical gloves under real surgical conditions. BMC Infect Dis.

[10] Pittet D, Allegranzi B, Boyce J (2009). World Health Organization world alliance for patient safety first global patient safety challenge Core Group of experts. The World Health Organization guidelines on hand hygiene in health care and their consensus recommendations. Infect Control Hosp Epidemiol.

[11] Peterson AF, Rosenberg A, Alatary SD (1978). Comperative evaluation of surgical scrub preparations. Surg Gynecol Obstet.

[12] Rotter ML, Kampf G, Suchomel M, Kundi M (2007). Population kinetics of the skin flora on gloved hands following surgical hand disinfection within 3 propanol-based hand rubs: a prospective, randomize, double-blinded trial. Infect Control Hosp Epidemiol.

[13] Partecke LI, Goerdt AM, Langner I, Jaeger B, Assadian O, Heidecke CD (2009). Incidence of microperforation for surgical gloves depends on duration of wear. Infect Control Hosp Epidemiol.

[14] Assadian O, Kramer A, Ouriel K, Suchomel M, McLaws ML, Rottman M (2014). Suppression of surgeons’ bacterial hand flora during surgical procedures using a new antimicrobial surgical glove. Surg Infect.

[15] European Norm (EN) 12791 (2009). Chemical disinfectants and antiseptics. Surgical hand disinfection – test method and requirement (phase 2/step 2).

[16] EN ISO/IEC 17025 (2005). General requirements for the competence of testing and calibration laboratories.

[17] Suchomel M, Weinlich M, Kundi M (2017). Influence of glycerol and an alternative humectant on the immediate and 3-hours bactericidal efficacies of two isopropanol-based antiseptics in laboratory experiments in vivo according to EN 12791. Antimicrob Resist Infect Control.

[18] Mangram AJ, Horan TC, Pearson ML, Silver LC, Jarvis WR (1999). Guideline for prevention of surgical site infection, 1999. Hospital infection control practices advisory committee. Infect Control Hosp Epidemiol.

[19] Laine T, Kaipia A, Santavirta J, Aarnio P (2004). Glove perforations in open and laparoscopic abdominal surgery: the feasibility of double gloving. Scand J Surg.

[20] Brough SJ, Hunt TM, Barrie WW (1988). Surgical glove perforations. Br J Surg.

[21] Kojima Y, Ohashi M (2005). Unnoticed glove perforation during thoracoscopic and open thoracic surgery. Ann Thorac Surg.

[22] Pitten FA, Herdemann G, Kramer A (2000). The integrity of latex gloves in clinical dental practice. Infection.

[23] Manjunath AP, Shepherd JH, Barton DP, Bridges JE, Ind TE (2008). Glove perforations during open surgery for gynaecological malignancies. BJOG.

